# Cardiac Troponin T (TNNT2) plays a potential oncogenic role in colorectal carcinogenesis

**DOI:** 10.1186/s12935-023-02977-9

**Published:** 2023-07-22

**Authors:** Yifan Liu, Ze Meng, Junqiang Niu, Le Tian, Yishan Chen, Qingju Meng, Yibing Liu, Zhiguo Zhou

**Affiliations:** 1 Hebei Medical University, Shijiazhuang, 050011 Hebei China; 2 The First Affiliated Hospital of Xingtai Medical College, Xingtai, 054000 Hebei China; 3 The Fourth Hospital of Hebei Medical University, 12 JianKang Road, Shijiazhuang, 050011 Hebei China

**Keywords:** Cardiac troponin TNNT2, Proliferation, Invasion, Metastasis, Mechanism of action

## Abstract

**Purpose:**

Colorectal cancer (CRC) is the third most common cancer in the world. The purpose of this study was to investigate the role of TNNT2 in the proliferation, migration and invasion of CRC cells and its expression in CRC tissues to better understand the regulatory role of TNNT2 in CRC.

**Methods:**

Western blotting (WB) and qPCR were used to detect the expression of TNNT2 in colorectal cancer tissues and paracancerous tissues. CCK-8, colony formation, Transwell and other experiments were used to clarify the role of TNNT2 in the proliferation, migration and invasion of colorectal cancer cells. Changes in TNNT2, EGFR and HER2 mRNA transcription levels were detected by SYBR Real-Time PCR assay, and the effects of TNNT2 overexpression or knockdown on the expression of EGFR, HER2 and EMT-related proteins in CRC cells were determined by WB. TNNT2 and EGFR intreaction was carried out in HCT116 cells by coimmunoprecipitation experiments.

**Results:**

The protein and mRNA expression level of TNNT2 in CRC tissues were higher than those in paracancerous tissues. The CCK-8 results suggested that overexpression of TNNT2 significantly promoted the proliferation of HCT116 and RKO cells, and TNNT2 konckdown gets the opposite result; and the colony formation results were the same as tthose of CCK-8 assay. Transwell invasion and migration experiments showed that overexpression of TNNT2 promoted the migration and invasion of HCT116 and PKO cells, and TNNT2 konckdown suppressed the migration and invasion of the these cells. The SYBR Green I real-time PCR method revealed that them RNA levels of TNNT2, EGFR and HER2 in the TNNT2 overexpression group were higher than those in RKO cells. WB showed that overexpressing TNNT2 increased the expression of EGFR and HER2 in HCT16 and RKO cells,decreased the expression of EMT marker E-cadherin, and increased the expression of Vimentin and N-cadherin. Konckdown of TNNT2 decreased the expression of EGFR and HER2, increased the expression of E-cadherin, and decreased the expression of Vimentin and N-cadherin in HCT16 and RKO cells. The immunocoprecipitation experiment showed that there was an interaction between EGFR and TNNT2.

**Conclusion:**

TNNT2 can promote the proliferation, invasion and metastasis of colorectal cancer cells. There is an interaction between TNNT2 and EGFR protein. TNNT2 can upregulate EGFR and HER2-related proteins in colorectal cancer cells and promote the occurrence of EMT. Therefore, TNNT2 can promote the invasion and metastasis of CRC cells through the EGFR/HER2/EMT signal axis, suggesting that TNNT2 is a potential target of CRC treatment.

Colorectal cancer (CRC) is the third most common cancer and the fourth most common cause of cancer-related death worldwide and presents a threat to human health [1, 2]. Epithelial-mesenchymal transition (EMT) is closely related to various important processes, such as tumor occurrence, progression, migration, intravascular infiltration and distant metastasis [3, 4]. EMT plays an important role in the distant metastasis of colorectal cancer. The occurrence, development and metastasis of colorectal cancer are also related to multiple gene mutations and the abnormal regulation of cell signal transduction. Epidermal growth factor receptor (EGFR) plays an important role in the occurrence and development of cancer [5]. The upregulation of EGFR (a member of the ErbB family) facilitates the occurrence and prognosis of colorectal cancer by activating multiple pathways (such as MAPK and PI3K pathways) [6].

The TNNT2 gene is located on chromosome 1q32 and encodes "cardiac troponin T2". It is highly expressed in the heart. TNNT2 is a gene that plays an important role in human heart muscle. Many studies have shown that TNNT2 is abnormally highly expressed in colorectal cancer and lung cancer, and is related to tumor grading and differentiation [7, 8]. However, the mechanism of TNNT2 in colorectal cancer is still unclear. We investigated the role of TNNT2 in the proliferation, migration and invasion of tumor cells, and the effect of TNNT2 on the biological behavior of colorectal cancer cells. Our results and the effect of TNNT2 on metastasis and the mechanism of TNNT2 in colorectal cancer are also discussed herein.

## Materials

The human colon cancer cell lines RKO and HCT116 are preserved by the Research Center of the Fourth Hospital of Hebei Medical University. Knockdown, overexpression RKO and HCT116 cells were constructed in the early stage of the research group, and they were all cultured in a constant temperature and humidity incubator (Panasonic). The culture conditions were DMEM medium with 37 ℃, 5% CO_2_ and 10% FBS. Colorectal cancer and paracancerous tissues were obtained from the Fourth Hospital of Hebei Medical University.

DMEM (Solarbio), a CCK-8 detection kit (Solarbio) and crystal violet staining solution (SolarBio) were used for cell culture. Transintro EL Transfection Reagent(Beijing full gold); siRNA and overexpression plasmid (Ji Ma gene); and Matrigel matrix adhesive(Shanghai Nova Company) were also used; The main antibodies were as follows: β-actin (BioCisco; 1:1000); E-Cadherin (R868) polyclonal antibody (Baaode; 1:1000); Vimentin (i444) polyclonal antibody (Baaode; 1:1000); N-Cadherin polyclonal antibody (Baaode; 1:1000); TNNT2 polyclonal antibody (BAODE; 1:1000); EGFR(E687) polyclonal antibody (Baaode; 1:1000); Erbb2/HER2 (K676) polyclonal antibody (Baaode; 1:1000). A fluorescent quantitative PCR instrument (CFX connect TM) was used.

## Methods

### Cell culture

RKO and HCT116 cells were cultured in DMEM medium containing 10% fetal bovine serum (FBS) at 37 ℃ and 5% CO_2_.

### Cell transfection

TNNT2-siRNA and overexpression plasmids were synthesized by Suzhou Ji Ma gene. Subsequently, 250 pmol si-RNA was added into 200 ul OPTI-MEM, gently mixed, and incubated for 5 min; Then 8 ul of TransIntroTM EL was added to the diluted si-RNA, gently mixed and incubated at room temperature for 20 min. Next, 4 ug of plasmid was added to 200 ul OPTI-MEM and mixed gently; followed by adding 8 ul TrasIntroTM EL to the diluted plasmid, mixed gently, and incubated at room temperature for 20 min. The mixed solution was added to the plate wells drop by drop, the plate was gently shaken to make the mixture uniform, and the mixtures were cultivated in an incubator containing 5% CO_2_ at 37 ℃ for 4 h; the medium was changed to complete to continue the culture, and detection was carried out after 48 h.

### RNA extraction and real-time PCR detection

The total RNA of cells and tissues was extracted with Trizol reagent (ambion) according to the instructions. 1 μl RNA was taken, and the quality and concentration of RNA were measured with an ultraviolet spectrophotometer to detect the integrity of RNA. Complementary DNA for reverse transcription was synthesized with HiFiScript gDNA Removal cDNA Synthesis Kit (Conway Century). Then real-time PCR analysis was carried out. With GAPDH as an internal reference, the expression level of TNNT2 was calculated by 2^−ΔΔCt^ method. TNNT2 primer sequence were as follows: forward, 5'-CGACGAGGGGAGAGAGAAAG-3'; reverse, 5'-ccgctgtcttctggatgtaacc-3'. Other primer were as follows:

HER-2-F(H) TGACTGCCTGTCCCTACAACTACC

HER-2-R(H) GCTGTGTTCCATCCTCTGCTGTC

EGFR-F(H) TACTTGGAGGACCGTCGCTTGG

EGFR-R(H) CTCTTCCGCACCCAGCAGTTTG

TNNT2-F(H) CGACGAGAGGAGGAGGAGAACAG

TNNT2-R(H) CCGCTCTGTCTTCTGGATGTAACC

### Western blot

Tissue homogenate and cells were lysed with RIPA lysis buffer on ice for 30 min, centrifuged at 12,000 r/min for 15 min, and the supernatant was collected. The protein concentration was detected by a BCA kit, and 30 μg of protein was subjected to 10% sodium dodecyl sulfonate-polyacrylamide gel electrophoresis. After electrophoretic separation, the protein was transferred to PVDF membrane, sealed with 5% skimmed milk powder for 1 h, washed by TBST, and incubated with the primary antibody for each target (1:2 000) and GAPDH(1:1 000) at 4 ℃ overnight, then, the second antibody (1:1 000) was added and incubated at room temperature for 1.5 h. ECL reagent was added to observe protein bands, β-Actin was used as an internal reference, and the grayscale values of the protein were calculated by Quantity One software.

### CCK8 and clone formation

After transfection, the cells were cultured for 4-6 h, digested and centrifuged and resuspended for inoculation into 96-well plates, with 100µL cell culture medium per well and 3 multiple wells for each sample, the cells were incubated in an incubator. 10 µL CCK-8 solution was added to each grouping well at four-time points (1d,2d,3d and4d) and incubated for 2 h in a CO_2_ incubator with a constant temperature of 37 ℃; and the absorbance at 450 nm was detected.

### Transwell

After washing away the residual cell culture medium with PBS, with the medium was replaced with RPMI-1640 medium for starvation culture overnight; trypsinized HCT116 and RKO cells were resuspended in serum-free RPMI-1640 medium, the cell density was adjusted to 1 × 10^6^ cells/mL, and 200 µL of the suspension was inoculated into the upper chamber of the Transwell. In both migration and invasion experiments, 800 µL medium containing 10% fetal bovine serum was added to the lower chamber, and Matrigel was precoated in the upper chamber of the Transwell in the invasion experiment. The cells were cultivated in a 37 ℃, 5% CO_2_ incubator for 24 h, the culture medium was removed from the transwell chamber, and the cells in the upper chamber were gently wiped off with a cotton ball. The cells that passed through to the lower chamber were fixed with 4% paraformaldehyde for 15 min, rinse with PBS 3 times for 5 min each; stained with Giemsa for 30 min, and rinsed with PBS 3 times for 3 min each. The cells were counted under the inverted microscope, and cell images of the inverted subventricular chamber were collected.

### Co-IP

Firstly, the total protein of HCT116 cells was extracted, and the protein concentration was determined to be 9.825 mg/ml. 10 uL of whole protein was taken as the input group, and the remaining protein sample was equally divided. Then, 1 μg of the corresponding antibody (IgG group plus IgG antibody, IP group plus TNNT2 antibody, EGFR antibody) was added, and the samples were incubated overnight at 4 ℃ on a shaker. 10 μL of protein A agarose beads was added to the protein extract, incubated with the antibody overnight and incubated on a shaker at 4 ℃ for 2–4 h, to couple the antibody to the protein A agarose beads for the immunoprecipitation experiment. The samples were centrifuged and washed 3 times at 4 ℃, 3000 rpm, Finally 2 × SDS loading buffer was added, the samples were boiled for 5 min, and WB was performed.

### Statistical analysis

Graphpad Prism 8.0.2 software was used for statistical analysis of experimental data, and the two groups of data were tested by Student's t-test. P < 0.05 indicated that the difference was statistically significant. All experiments are repeated independently 3 times.

## Results

### Expression of TNNT2 in colorectal cancer

**Fig. 1:**
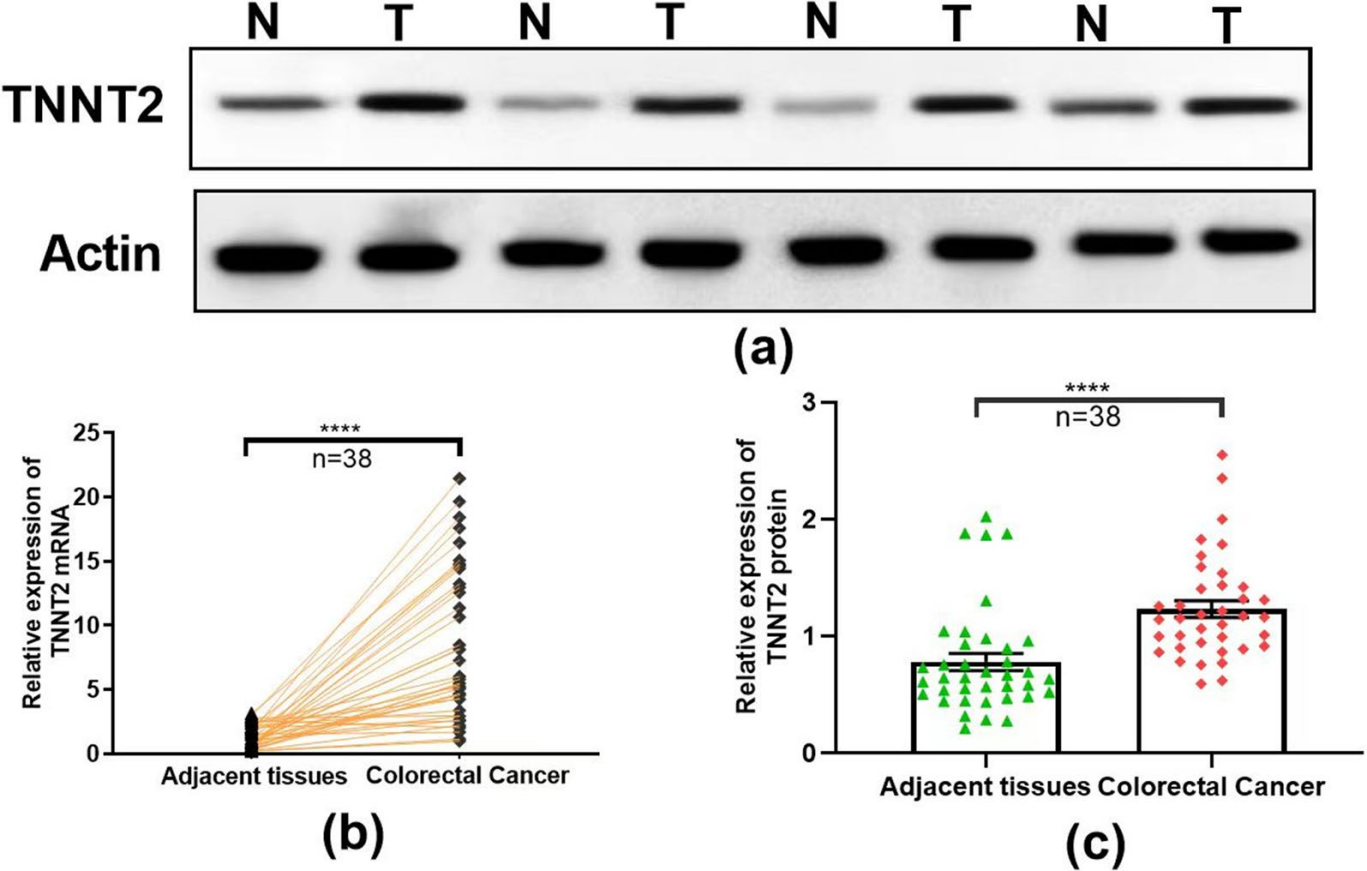
Expression of TNNT2 protein and mRNA in colorectal and adjacent tissues. **a**, **c** Western blotting shows the TNNT2 protein levels in the colorectal and adjacent tissues from 38 patients. **b** qPCR indicates the TNNT2 mRNA levels in the colorectal and adjacent tissues from 38 patients. ****P < 0.0001, difference with adjacent tissues

38 colorectal cancer tissues and paraneoplastic tissue were collected, and the protein expression of TNNT2 in cancer tissues and adjacent tissues was detected by Western blotting (Fig. 1a). qPCR was used to detect the mRNA expression of TNNT2 in cancer tissues and paraneoplastic tissue (Fig. 1b). The results suggest that the expression of TNNT2 in colorectal cancer tissues is higher than that in paraneoplastic tissue.

### Effects of TNNT2 overexpression and knock-down on the proliferation of colorectal cancer cells in vitro

**Fig. 2:**
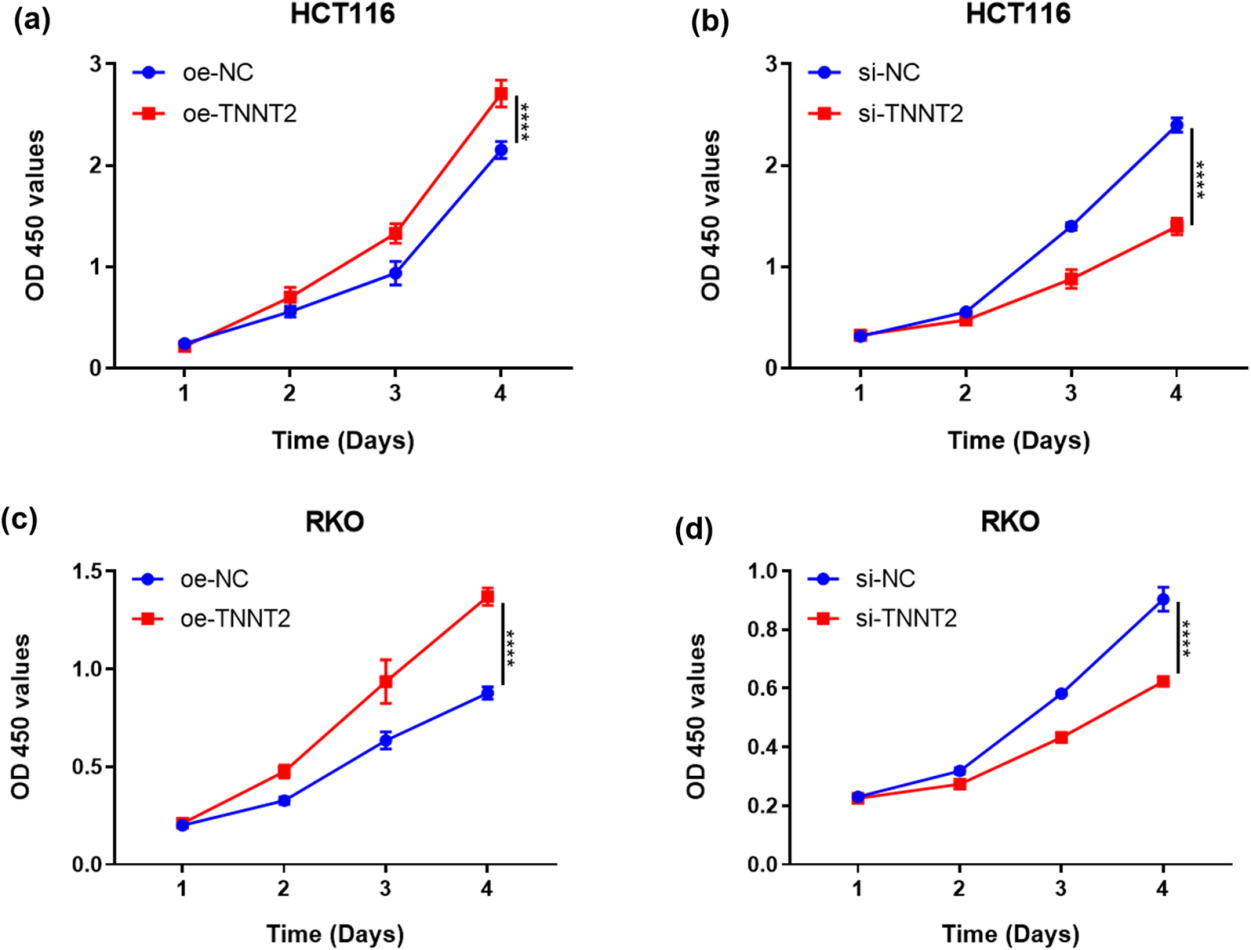
CCK-8 assay of HCT116 and PKO transfected with oe-TNNT2 and si-TNNT2, respectively. **a** CKK-8 assay shows that after 4 days of cultivation, the OD450 value in oe-TNNNT2 HCT116 cells was significantly higher than that in the control group;**b** CKK-8 assay showed OD450 values were lower of HCT116 cells of si-TNNT2 than control group; **c** CKK-8 assay indicated OD450 values were higher in PKO cells of oe-TNNT2 than control group; **d** CKK-8 assay showed OD450 values was lower of PKO cells of si-TNNT2 than control group. *** *P < 0.0001, difference with control group in blue and green puncta. *Oe-NC* Overexpression control group, *oe-TNNT2* Overexpression TNNT2 group, *si-NC* Konck-down control group, *si-TNNT2*, Knock-down TNNT2

CCK-8 assays showed that TNNT2 overexpression significantly promoted the proliferation of HCT116 and RKO cells (Fig. 2a, c). Notably, TNNT2 maintained its ability to promote the proliferate colorectal cancer cells after a long time. For example, the proliferation rate of HCT116 and RKO cells was different between TNNT2-overexpressing group and the control group after 96 h (P = 0.0020 and P < 0.0001), and the difference increased gradually. Knockdown of TNNT2 inhibits cell proliferation in HCT116 and RKO cells (Fig. 2b, d).

**Fig. 3:**
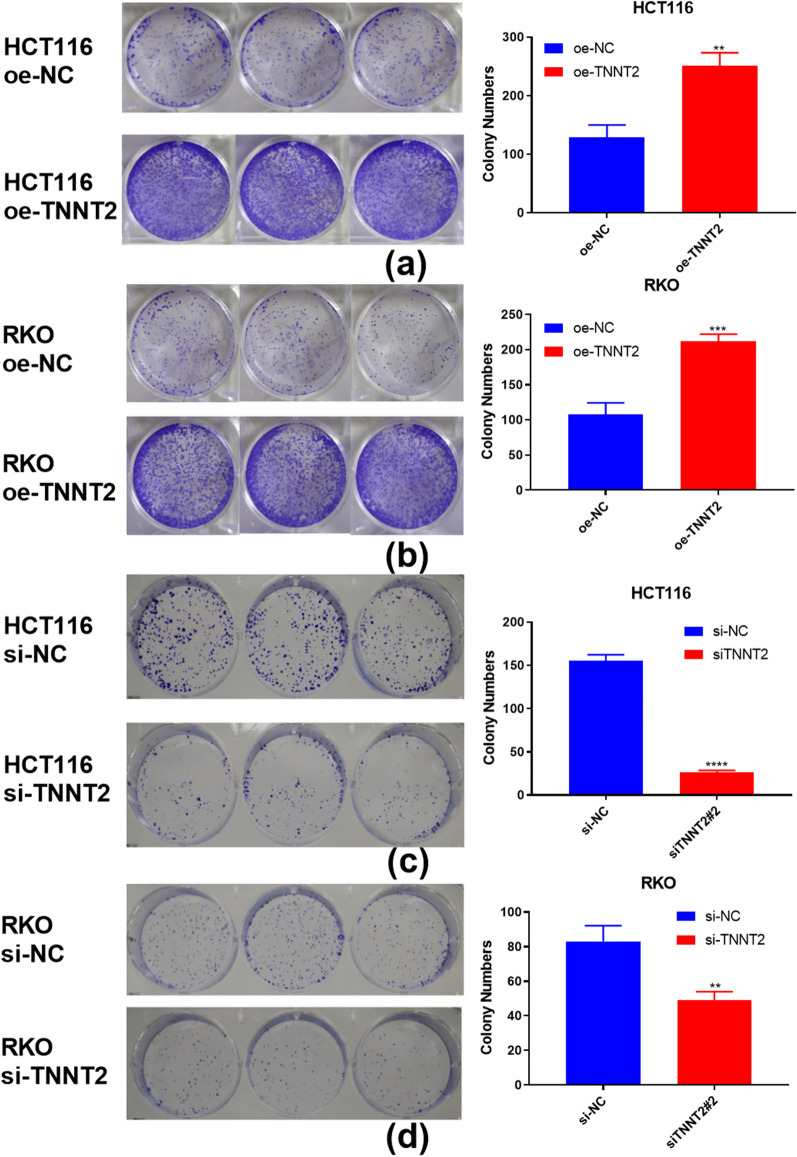
Colony formation assay of HCT116 and PKO transfected with oe-TNNT2 and si-TNNT2, respectively. **a**,**b** The colony formation assay showed that colony number is increased in HCT116 and RKO cells of oe-TNNT2 group compared with those of the control group; **c** Colony formation assay showed colony number was greatly decreased in HCT116 cells of siTNNT2 group compared with those of the control group.**d** Colony formation assay indicated that colony number is decreased in RKO cells of the si-TNNT2 group compared with those of the control group. **p < 0.01, ***p < 0.001, ****p < 0.0001, difference with control group in blue puncta. *Oe-NC* Overexpression control group, *oe-TNNT2*:Overexpression TNNT2 group *si-NC*: Konck-down control group, *si-TNNT2* Knock-down TNNT2

The tumorigenic ability of cells infected with lentivirus is suggested by the ability of cells to form colonies on cell culture plates after infection. The results showed that the number of clonies formed by overexpression of TNNT2 in HCT116 (P = 0.0023) and RKO (P = 0.0008) cells was significantly higher than that in the control group (Fig. 3a, b). TNNT2 knockdown can inhibit the clonogenic ability of colorectal cancer cells (Fig. 3c, d).

### Effects of overexpression and knockdown of TNNT2 on the migration ability of HCT116 and RKO cells

**Fig. 4:**
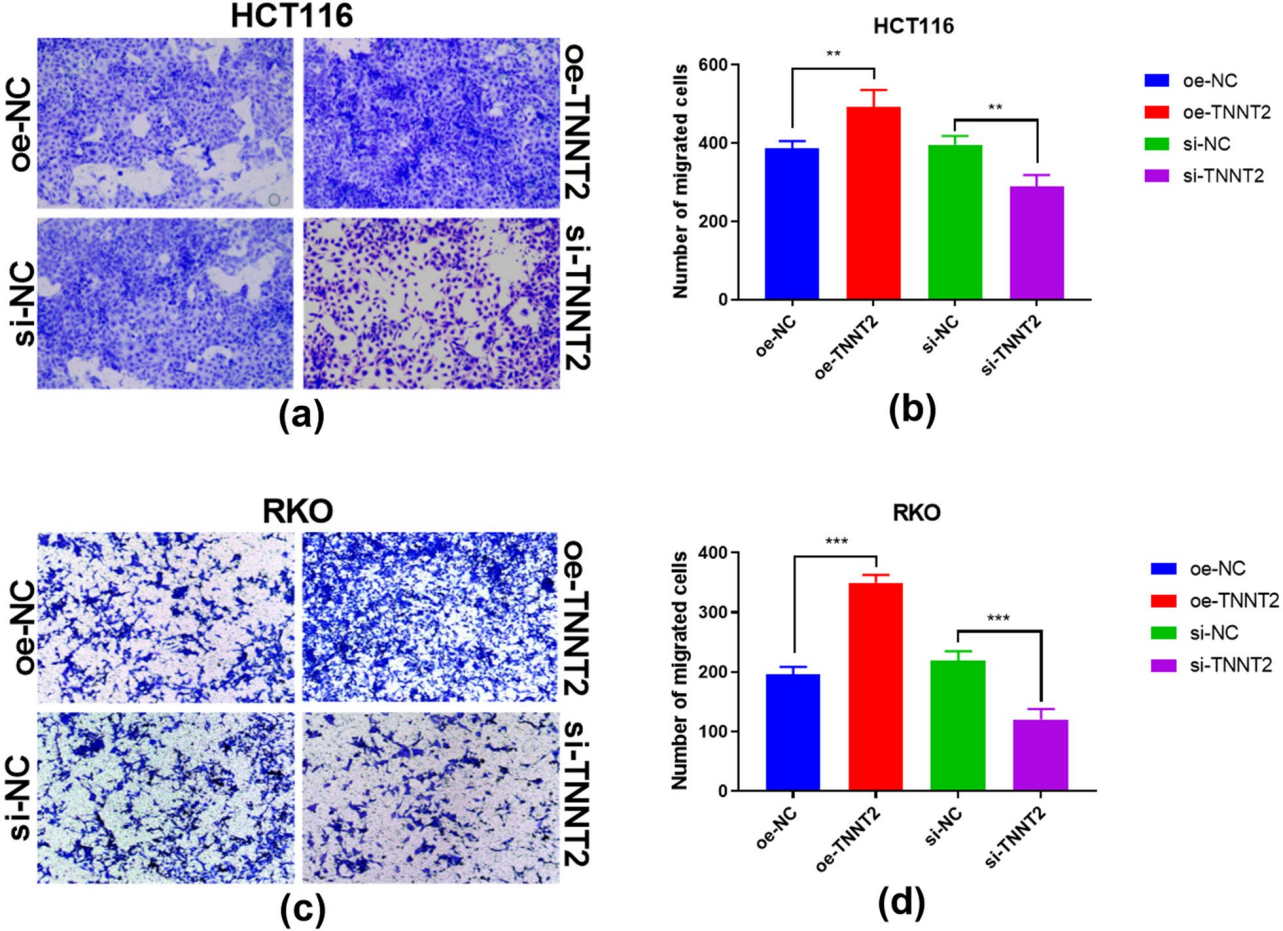
Migration ability of HCT116 and RKO cells with overexpression and knockdown. Transwell assay shows cell migration ability of HCT116 cells in the oe-TNNT2 and si-TNNT2 **a**, **b** and **c**, **d** groups. ** P < 0.01, difference with control group in blue and green puncta. **p < 0.01, ***p < 0.001, difference with control group in blue and green puncta. *Oe-NC* Overexpression control group, *oe-TNNT2*:Overexpression TNNT2 group, *si-NC* Konck-down control group, *si-TNNT2*: Knock-down TNNT2

In the migration experiment, the number of cells entering the lower chamber in the TNNT2 overexpression group was significantly higher than that in the negative control group, while the number of cells entering the lower chamber in the TNNT2 knockdown group was significantly less than that in the negative control group (Fig. 4a, b). There were differences between the oe-NC group and the oe-TNNT2 group in HCT116 and RKO cells, and the cell migration ability of the oe-TNNT2 group was greater than that of the oe-NC group. There were differences between the si-NC group and si-TNNT2 group. The cell migration ability of the si-TNNT2 group was lower than that of the si-NC group, and the differences were statistically significant (Fig. 4c, d).

### Effects of overexpression and knockdown of TNNT2 on the invasion ability of HCT116 and RKO cells

**Fig. 5:**
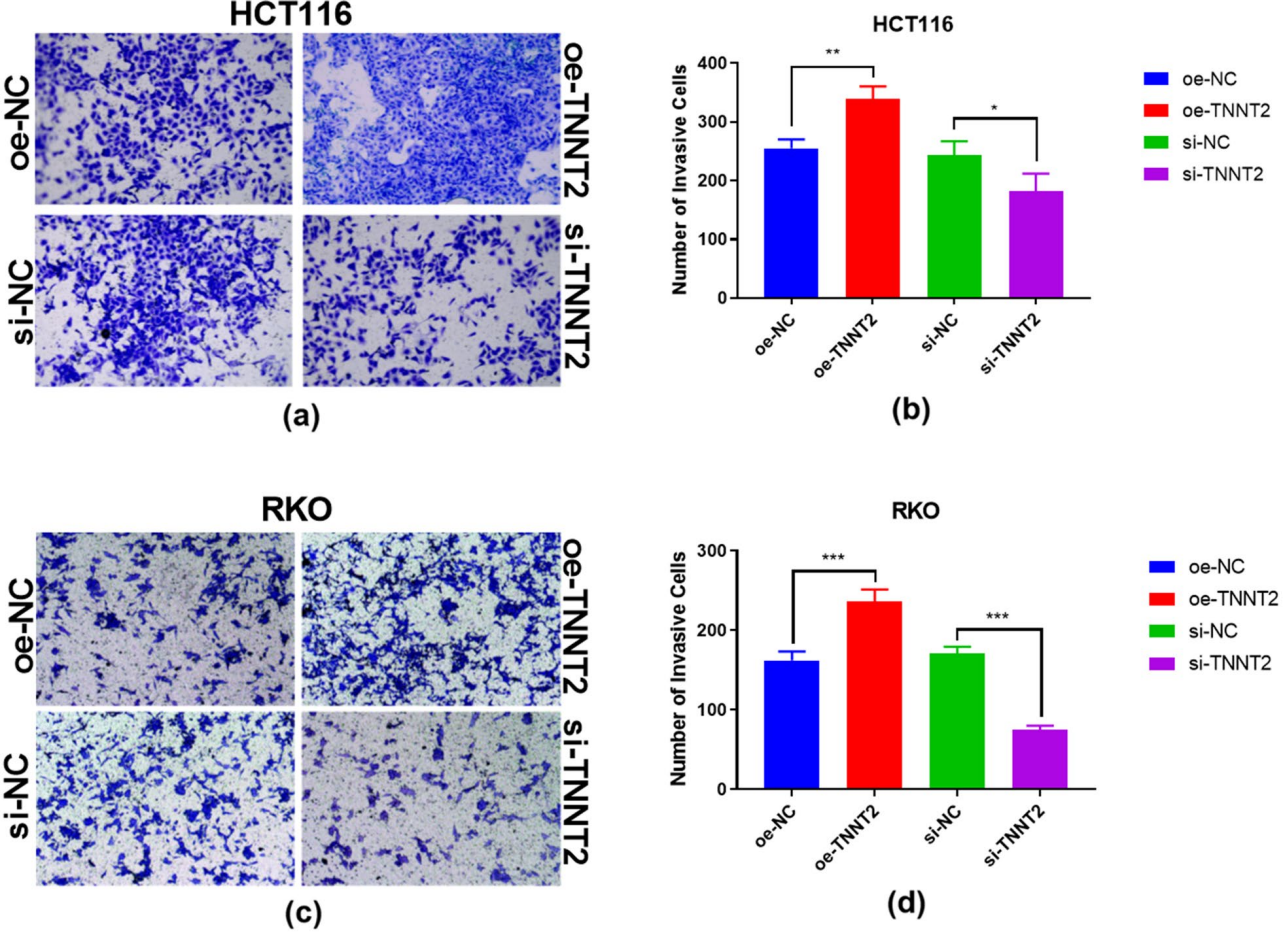
Invasion ability of HCT116 and RKO cells with TNNT2 overexpression and knock-down. Transwell assay shows cell invasion ability of oe-TNNT2 and si-TNNT2 of HCT116 cells (**a**, **b**) and (**c**, **d**). *p < 0.05, **p < 0.01, ***p < 0.001, difference with control group in blue and green puncta. **P < 0.05, ** P < 0.01, *** P < 0.001, difference with control group in blue and green puncta. *Oe-NC* Overexpression control group, *oe-TNNT2* Overexpression TNNT2 group, *si-NC*: Konck-down control group, *si-TNNT2*: Knock-down TNNT2

In the invasion experiment, the number of cells entering the lower chamber in the TNNT2 overexpression group was significantlygreater than that in the negative control group, while the number of cells entering the lower chamber in the TNNT2 knockdown group was significantly less than that in the negative control group (Fig. 5). There were differences between the oe-NC group and the oe-TNNT2 group in HCT116 and RKO cells, and the cell invasion ability of the oe-TNNT2 group was greater than that of the oe-NC group. There were differences between the si-NC group and the si-TNNT2 group. The cell invasion ability of the si-TNNT2 group was lower than that of the si-NC group, and the differences were statistically significant (Fig. 5).

### The transcription levels of TNNT2, EGFR and HER2 mRNA in HCT116 and RKO cells were detected by SYBR GreenI real time PCR

**Fig. 6:**
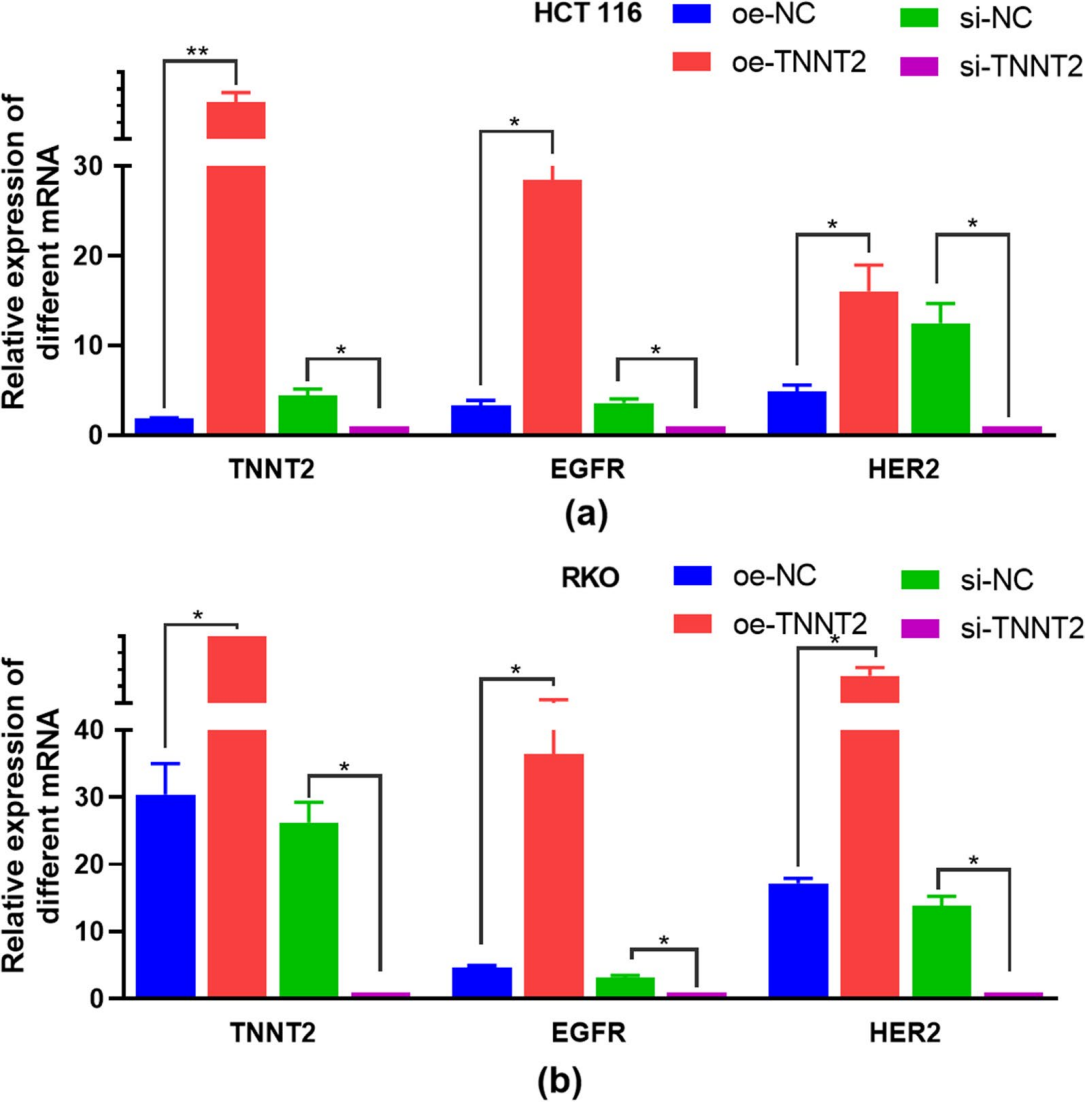
Expression of TNNT2, EGFR and HER2 in HCT116 and PKO cells. **a** qPCR assays of the RNA levels of TNNT2, EGFR and HER2 in oe-TNNT2 and si-TNNT2 of HCT116 cells **a** and RKO cells (**b**). *p < 0.05, **p < 0.01, difference with control group in blue and green puncta. *Oe-NC* Overexpression control group, *oe-TNNT2* Overexpression TNNT2 group, *si-NC* Konck-down control group, *si-TNNT2* Knock-down TNNT2

In HCT116 and RKO cells, the transcription levels of TNNT2, EGFR and HER2 mRNA in the oe-TNNT2 group were generally higher than those in the si-TNNT2 group. The transcript levels were generally higher in RKO group (Fig. 6).

### Western blotting of the expression of EGFR, HER2 and EMT-related proteins in HCT116 and RKO cells of colorectal cancer cells with TNNT2 overexpression or knockdown

**Fig. 7:**
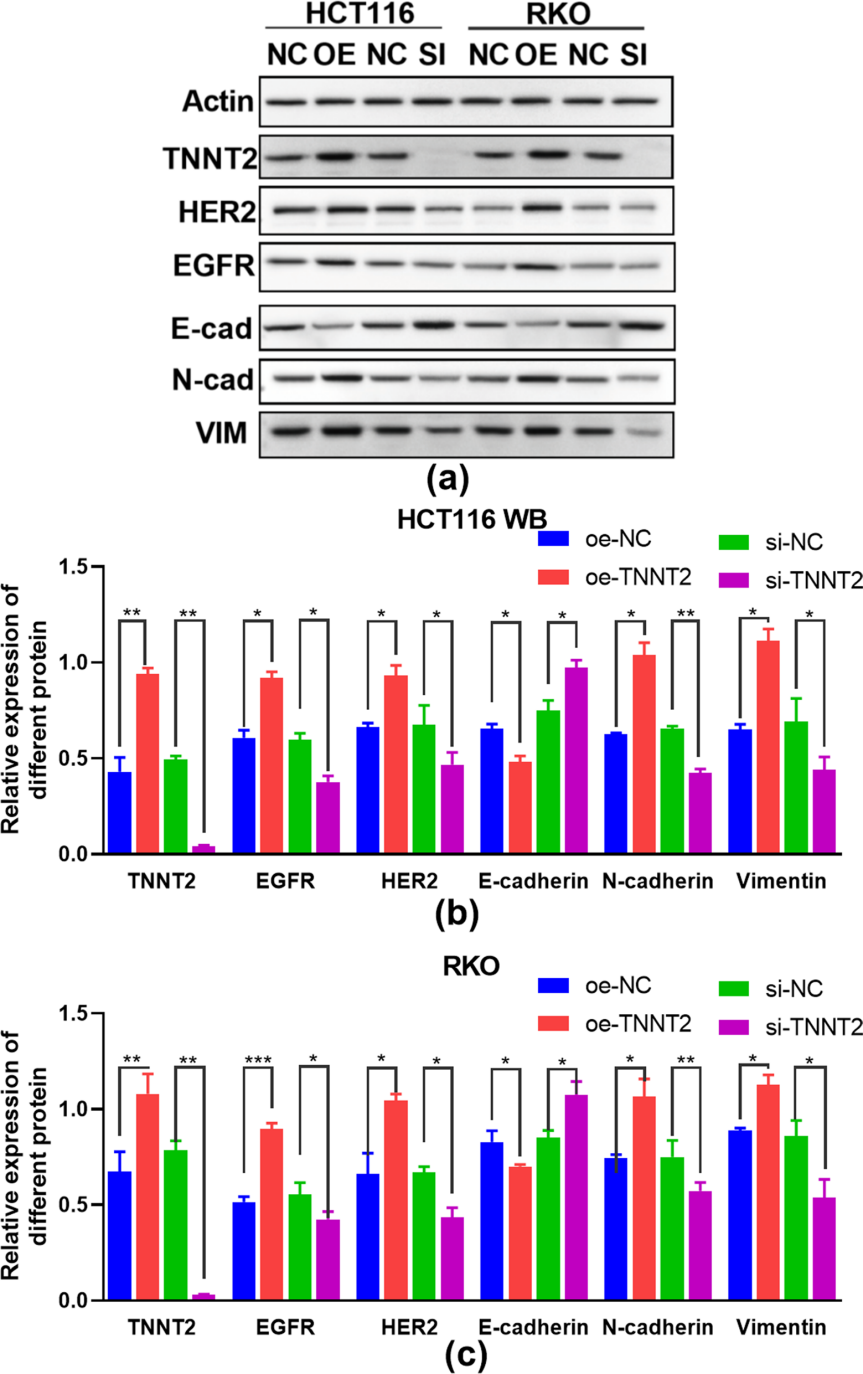
Expression of TNNT2, EGFR, HER2, E-cadherin, N-cadherin and Vimentin of HCT116 and RKO cells. Western blot assay shows the protein levels of TNNT2, EGFR, HER2, E-cadherin, N-cadherin and Vimentin in oe-TNNT2 and si-TNNT2 of HCT116 cells **a**, **b** and RKO cells **a**, **c**. *p < 0.05, **p < 0.01, ***p < 0.001, difference with control group in blue and green puncta. Oe-NC: Overexpression control group; *oe-TNNT2* Overexpression TNNT2 group, *si-NC* Konck-down control group, *si-TNNT2* Knock-down TNNT2

Overexpression of TNNT2 increased the expression of EGFR and HER2 in HCT16 and RKO cells (Fig. 7b,c). However, knocking down TNNT2 decreased the expression of EGFR and HER2 in HCT16 and RKO cells (Fig. 7b,c). E-cadherin can maintain the epithelial characteristics of cells by regulating various signal pathways [9]. As shown in Figures 7b and c, E-cadherin was downregulated when TNNT2 was overexpressed and up-regulated when TNNT2 was knocked down, and the differences were significant (P<0. 05). On contrast, the expressions of vimentin [10] and N-cadherin [11], which are highly related to the mesenchymal characteristics of cells, increased significantly when TNNT2 was overexpressed but decreased when TNNT2 was knocked down, and the differences were statistically significant (P<0. 05). The above results indicate that overexpression of TNNT2 can promote EMT of colorectal cancer cells, while knockdown of TNNT2 has the opposite effect. The overexpression of TNNT2 can inhibit the expression of EMT marker E-cadherin and promote the expression of Vimentin and N-cadherin.

### Verification of the Interaction between TNNT2 and EGFR by Immunoprecipitation

**Fig. 8:**
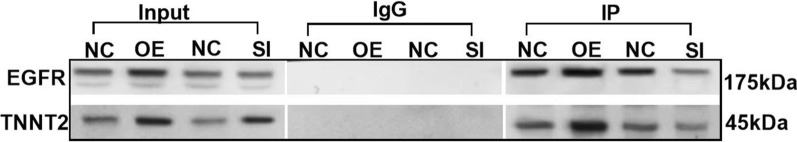
Co-IP analysis of EGFR and TNNT2 in HCT116 cells. The proteins analyzed and sizes of molecular weight standards are indicated on the left and right, respectively, and by arrows when necessary (IP, immunoprecipitation). OE: High expression group; KD: Low expression group. Input group:TNNT2 and EGFR proteins are expressed in the sample and can be detected by WB;lgG group:negative control; IP group: The precipitation experiment was carried out by using the antibody of protein

Total protein extracts isolated from HCT116 cells. Co-IP experiments can identify proteins via direct or indirect interactions or in a protein complex. Here, we detected TNNT2 and EGFR interaction by this method.Coprecipitated proteins were identified by western blotting. Co-IP was also performed with antibody against IgG as a negative control. The interaction between TNNT2 and EGFR was verified by Immunoprecipitation experiment in the HCT116 cell line (Fig. 8).

## Discussion

Normal colon tissue consists of smooth muscle. Previous studies have shown that the expression of TNNT2 is different between CRC tumors and normal tissues. TNNT2 is expressed at a low level in normal colon tissues but is significantly upregulated in colorectal cancer tissues [7]. Our study showed that there was a significant difference in the expression of TNNT2 between colorectal cancer tissue and paraneoplastic tissue, and TNNT2 was highly expressed in colorectal cancer tissue and expressed at low levels in paraneoplastic tissues. Furthermore, our studies have shown that TNNT2 can promote the growth, colony formation and migration of colorectal cancer cells, inhibit cell apoptosis and aging, and play a potential role in promoting colorectal cancer. Therefore, TNNT2 may be an oncogene in colorectal cancer.

The occurrence and development of CRC are related to many gene mutations and the abnormal regulation of cell signal transduction, and among the related factors, EGFR plays an important role in the occurrence and development of tumors [5]. EGFR can activate a variety of downstream signaling pathways, which can induce subsequent biological effects, participate in the occurrence and development of colon cancer, and may affect tumor metastasis and prognosis [12]. C-erbB-2 is a cell-derived proto-oncogene and a member of the EGFR family. After activation, it can participate in the regulation of cell growth, proliferation, division and apoptosis through different signal transduction pathways [5, 13]. Some studies have confirmed that the amplification degree of C-erbB-2 in CRC is higher than that in normal colorectal mucosa. The lower the grade of CRC is, the stronger the amplification of C-erbB-2, so the malignant transformation of CRC and its biological behavior depend on the activation of C-erbB-2 [1, 5, 14]. In our study, PCR experiments showed that the transcription levels of TNNT2, EGFR and HER2 mRNA in TNNT2-overexpressing HCT116 and RKO cells were generally higher than those of TNNT2-konckdown in HCT116 and RKO cells. WB experiment showed that TNNT2 increased the expression of EGFR and HER2-related proteins in HCT16 and RKO cells, while knocking down TNNT2 decreased the expression of EGFR and HER2-related proteins in HCT16 and RKO cells. CO-IP experiments showed that EGFR and TNNT2 interacted.

Our study confirmed TNNT2 overexpression inhibits the expression of EMT marker E-cadherin and promotes the expression of Vimentin and N-cadherin. However, knocking down TNNT2 increased the expression of E-cadherin, and decreased the expression of Vimentin and N-cadherin. EMT plays an important role in the distant metastasis of colorectal cancer. EMT usually occurs during development and wound healing in healthy individuals, but it is closely related to the occurrence, progression, migration, intravascular infiltration and distant metastasis of tumors, as it weakens the adhesion between cells and enhances the invasion of tumor cell [3, 4]. E-cadherin plays an important role in tumor cells morphology and structural integrity, and in tumor cell migration and invasion [9]. N-cadherin has the opposite effect to E-cadherin in the malignant transformation, invasion and migration of malignant tumor cells, it promotes the separation of cancer cells from cancer tissues and their adhesion to normal cells [15]. Vimentin is an interstitial markers, that is expressed in many kinds of cells. The increase in its expression level indicates that the epithelial-mesenchymal transition of cells is enhanced, which plays an important role in the distant metastasis of epithelial fine carcinoma [16]. Therefore, TNNT2 expression can promote EMT.

In summary, this study found that TNNT2 can promote the proliferation, invasion and metastasis of colorectal cancer cells through CCK-8, colony formation experiment and Transwell assays. PCR, WB and Co-IP experiments revealed an interaction between TNNT2 and EGFR protein. TNNT2 overexpression upregulated EGFR- and HER2-related proteins in colorectal cancer cells and promoted the occurrence of EMT(represented by changes in E-cadherin, vimentin and N-cadherin expression), which led to the loss of polarity of colorectal cancer epithelial cells, decreased cell adhesion, reduced contact with surrounding cells and stromal cells, and reduced cell-to-cell interaction. This study reveals that TNNT2 can promote the invasion and metastasis of colorectal cancer through EGFR/HER2/EMT signal axis, and reveals the potential of TNNT2 as a therapeutic target for colorectal cancer.

## Data Availability

All data generated or analyzed during this study are included in this published article and its supplementary information files.
