# Correction: Cardiac Troponin T (TNNT2) plays a potential oncogenic role in colorectal carcinogenesis

**DOI:** 10.1186/s12935-023-03127-x

**Published:** 2023-12-19

**Authors:** Yifan Liu, Ze Meng, Junqiang Niu, Le Tian, Yishan Chen, Qingju Meng, Yibing Liu, Zhiguo Zhou

**Affiliations:** 1 Hebei Medical University, Shijiazhuang, 050011 Hebei China; 2 The First Affiliated Hospital of Xingtai Medical College, Xingtai, 054000 Hebei China; 3 The Fourth Hospital of Hebei Medical University, 12 JianKang Road, Shijiazhuang, 050011 Hebei China

Correction to: Cancer Cell International (2023) 23:146

10.1186/s12935-023-02977-9

In this article [1], the order that the authors appeared in the author list was incorrect, the order of co-authors Yibing Liu and Zhiguo Zhou were inadvertently transposed.

The original article has been corrected.
